# Human retinotopic mapping: From empirical to computational models of retinotopy

**DOI:** 10.1167/jov.25.8.14

**Published:** 2025-07-16

**Authors:** Fernanda L. Ribeiro, Noah C. Benson, Alexander M. Puckett

**Affiliations:** 1 School of Electrical Engineering and Computer Science, The University of Queensland, Brisbane, Queensland, Australia; 2 Department of Medicine, Justus-Liebig University Giessen, Giessen, Germany; 3 School of Psychology, The University of Queensland, Brisbane, Queensland, Australia; 4 Queensland Brain Institute, The University of Queensland, Brisbane, Queensland, Australia; 5 eScience Institute, University of Washington, Seattle, WA, USA; 6 Graduate School of Health, University of Technology Sydney, Ultimo, New South Wales, Australia

**Keywords:** vision, retinotopic organization, early visual cortex, V3, interindividual variability, fMRI

## Abstract

The visual cortex encodes the visual field through numerous bilaterally paired, topologically organized two-dimensional maps along the cortical surface. Although these representations exhibit a largely consistent organization across individuals, substantial interindividual variability exists in both the structure and functional organization of the visual cortex. To better characterize this variability, researchers have increasingly turned to computational approaches, alongside empirical methodologies and encoding models, to predict individual-level retinotopic organization. As these advances continue to shape the study of retinotopic organization, it is crucial to revisit the methodologies used to generate and model these maps. In this review, we examine empirical and theoretical work aimed at reconstructing and modeling visual field maps in the human visual cortex. Specifically, we discuss how empirical retinotopic mapping has facilitated the development of theoretical and computational models of retinotopy and, in turn, how these models have enhanced our understanding of the retinotopic organization of the human visual cortex. Finally, we outline a non-exhaustive set of future directions for leveraging models of retinotopy to further investigate structure–function relationships, interindividual variability, and more.

## Introduction

A common feature of the sensory cortex is the representation of sensory input as two-dimensional (2D) maps along the cortical surface. In the visual cortex, the visual field is represented by numerous bilaterally paired 2D maps that preserve the topology of each visual hemifield (or quarter visual field) in the contralateral hemisphere. That is, adjacent neurons in the visual cortex represent adjacent locations in the visual field and retina. These representations of the visual field in the brain, known as retinotopic or visual field maps, exhibit a largely consistent organization across individuals. The topological representation of the visual field within these maps, as well as their adjacency, follows a common pattern. However, despite this overall consistency, significant interindividual variability exists in both the structure and functional organization of the visual cortex across humans. For example, retinotopic maps vary significantly in size, the cortical territory they occupy, and their topological organization (Amunts, Malikovic, Mohlberg, Schormann, & Zilles, 2000; Benson et al., 2022; Dougherty et al., 2003; Gilissen et al., 1995; Gilissen & Zilles, 1995; Gilissen & Zilles, 1996; Hasnain, Fox, & Woldorff, 1998; Rademacher, Caviness, Steinmetz, & Galaburda, 1993; Ribeiro et al., 2023; Stensaas, Eddington, & Dobelle, 1974).

To better characterize this variability, researchers have increasingly relied not only on empirical methodologies and encoding models, but also on computational approaches to predict individual-level retinotopic organization. Models of retinotopy—mathematical and computational frameworks designed to describe and predict retinotopic organization—have become an essential tool in this effort. Over the past decades, the development of these models has been accelerated by advances in computational methods and the availability of large public datasets containing retinotopic maps (Allen et al., 2021; Benson et al., 2018; Hebart et al., 2023; Himmelberg et al., 2021; Pinho et al., 2024; Sengupta et al., 2016). As these advances continue to shape the study of retinotopic organization, it is essential to revisit the methodologies used to generate and interpret these maps.

Here, we briefly review the history and methodologies of retinotopic mapping—the process of acquiring retinotopic maps at the individual level through empirical data or computational models. Although empirical retinotopic mapping remains foundational and is discussed in this review, our primary focus is on exploring recent advances in modeling the retinotopic organization of the human visual cortex and its implications for understanding interindividual variability in retinotopic organization and uncovering its links to human perception. In doing so, this review not only captures the current state of the field, but also highlights exciting future possibilities.

## A brief historical overview of human retinotopic mapping

During the late 19th century and the beginning of the 20th century, several investigations regarding the functional divisibility of the cerebral cortex led to the theory that specific areas in the brain encode visual experiences (for a detailed account of these historical events, please refer to Zeki [1993]; briefer accounts are available in Glickstein & Whitteridge [1987] and Carlson & Devinsky [2009]). Among these, Munk's (1881) research was particularly pivotal—he demonstrated that removing the occipital lobe of one hemisphere of a dog's brain induced hemianopia (loss of sight of a visual hemifield) of the contralateral visual field, providing the first evidence of the localization of visual experiences in non-human mammals. Building on this knowledge, research between 1890 and the early 1900s further refined the understanding of cortical visual processing, leading to the development of the concept of the cortical retina, which posits that a topological representation of the visual field is mapped in the striate cortex (Fishman, 1997). Salomon Henschen's *post-mortem* examination of hemianopia cases (Henschen, 1890) revealed that lesions consistently occurred in the striate cortex (also known as the primary visual cortex or V1). Next, in more fine-grained analyses, Tatsuji Inouye, Gordon Holmes, and William Lister evaluated vision impairments in soldiers who suffered brain injuries during the Russo-Japanese War (1904–1905) (Inouye, 1909) and World War I (1914–1918) (Holmes & Lister, 1916). By examining entry and exit points of bullet wounds (Inouye, 1909) or using radiographs (Holmes & Lister, 1916), they identified cases where damage to specific cortical regions corresponded to distinct patterns of visual field loss.

**Figure 1.**
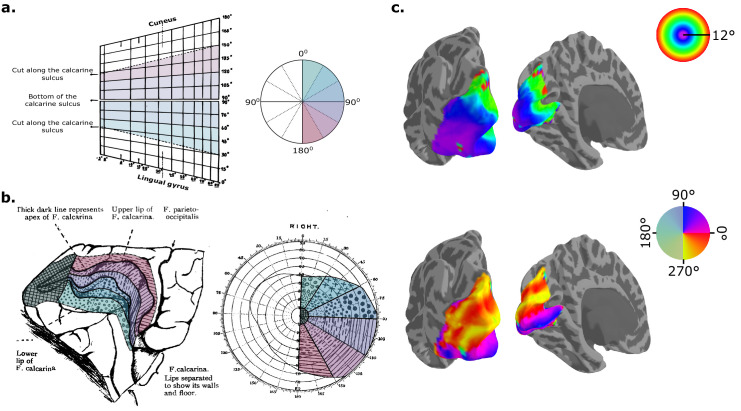
Diagrams of the visual field representation in the primary visual cortex. (**a**) Inouye's diagram shows how different portions of the right visual hemifield (right) are represented in the left hemisphere's primary visual cortex (left). As per his diagram, the brain region between the Cuneus and Lingual gyrus is represented as a gridded plane with vertical lines representing varying eccentricities (distance away from the center of the visual field) and horizontal lines representing different polar angles (or clock position). The figure was adapted from Inouye (1909). (**b**) A similar diagram was proposed by Holmes with a schematic medial view of the visual cortex (left). In this diagram, the visual field is represented as a dartboard, accompanied by the visual field projection in the primary visual cortex, with circular contours at regular radii marking the visual eccentricities and radial lines indicating the polar angles. The figure was adapted from Holmes (1918). (**c**) A contemporary representation of the right visual hemifield across early visual areas (V1, V2, and V3) in the left hemisphere. Using the polar coordinate system, the distance away from the center of the visual field (top) and the angle (bottom) are represented separately. Here, we can clearly observe that the representation of the center of vision spans the lateral surface across early visual areas, including V1.

These studies provided critical insights into the organization of the visual cortex. They established that 1) the occipital pole represents the center of vision and that the anterior portion of the calcarine sulcus represents the periphery; 2) the horizontal meridian is represented deep inside the calcarine sulcus, with the lower visual field represented in the upper (dorsal) bank of the sulcus and the upper visual field represented in the lower (ventral) bank; and 3) more cortex is dedicated to representing the center of vision than the periphery—a property that was later confirmed in non-human primates (Daniel & Whitteridge, 1961; Talbot & Marshall, 1941) and termed cortical magnification. Inouye proposed the first reasonably accurate schematic of the visual field projection to the primary visual cortex (Figure 1a), but Holmes later introduced a more intuitive diagram (Holmes, 1918) (Figure 1b), which remains widely used to visualize contemporary maps and models more than a century later (Figure 1c). Despite some inaccuracies, these early findings—particularly the cortical retina concept and the detailed topography of visual field representation—laid the foundation for contemporary vision research and the development of computational models capable of predicting visual field projections onto the cortex.

Inouye, Holmes, and Lister's contributions were remarkably ahead of their time, predating the next significant breakthroughs by approximately 70 years. A key milestone came in 1986, when the first retinotopic mapping study in healthy volunteers used positron emission tomography—an imaging technique that employs radiotracers to monitor changes in blood flow—to reveal systematic variations in brain activity corresponding with visual stimuli (Fox et al., 1986; Fox, Miezin, Allman, Van Essen, & Raichle, 1987). Around the same time, Horton and Hoyt (1991) leveraged the superior spatial resolution of structural magnetic resonance imaging (MRI) to further investigate cortical magnification, a principle initially identified in early lesion studies but now quantified with greater precision. Their findings confirmed that more cortical territory is dedicated to central vision than the periphery, reinforcing the concept established decades earlier by Holmes and his predecessors. Notably, however, they found that Holmes’ original map underestimated the degree of foveal magnification, highlighting the importance of modern neuroimaging techniques in refining our understanding of visual field representation.

Building on earlier breakthroughs, the advent of functional MRI (fMRI) marked a pivotal moment in retinotopic mapping, enabling noninvasive measurement of induced brain activity at an unprecedented spatial resolution. By the early 1990s, researchers had paved the way for this advent by establishing that local neuronal activity could be inferred from changes in blood oxygenation levels (Buxton, 2013; Fox & Raichle, 1986; Ogawa, Lee, Kay, & Tank, 1990). These major discoveries in turn led to independent studies by DeYoe, Bandettini, Neitz, Miller, and Winans (1994), Engel et al., (1994), and Sereno et al. (1995) that demonstrated how a more precise and systematic mapping of visual processing areas could be obtained using fMRI.

All three labs used phase-encoded designs (Bandettini et al., 1993) to systematically map the retinotopic organization of the human visual cortex using fMRI. At Stanford University, Engel et al. (1994) sought to determine whether smooth eccentricity maps could be observed along the calcarine sulcus by parametrically varying a specific aspect of a visual stimulus. Their design used high-contrast flickering checkerboard patterns inside of ring apertures that expanded continuously over time, allowing them to track the traveling wave of blood oxygenation level-dependent activity from the posterior to the anterior calcarine sulcus—a progression corresponding with the cortical representation of foveal to peripheral visual field locations—demonstrating that fMRI could resolve fine-scale functional organization in V1 with just 192 seconds of scanning. Meanwhile, DeYoe et al. (1994) at the Medical College of Wisconsin used a similar phase-encoded approach, but rather than mapping eccentricity, they focused on retinotopic representation along the polar angle axis, a method they referred to as phase tagging. Their findings not only confirmed fMRI's ability to capture V1 retinotopy, but also extended these observations to the extrastriate cortex, reinforcing its potential for broader functional imaging applications. Around the same time, at the University of California, Sereno et al. (1995) used phase-encoded mapping to identify multiple visual field maps beyond V1, including areas V2, V3, and even a couple higher-order visual regions. Collectively, these studies established phase-encoded fMRI as a robust and efficient method for mapping human visual field representation, setting the stage for its widespread adoption in vision science.

## Modern approaches to measuring retinotopic maps

With phase-encoded fMRI firmly established as a powerful tool for mapping visual field representation, subsequent advancements have further refined measurement techniques, enhancing spatial precision and extending coverage beyond early visual areas. Modern approaches build on these foundations, introducing paradigms that yield more detailed and individualized estimates of retinotopic organization. These innovations have not only improved spatial accuracy, but also broadened the applications of retinotopic mapping. In the following section, we explore these contemporary techniques and their contributions to advancing our understanding of visual processing.

### Population receptive field (pRF) modeling

pRF modeling, a methodological succession of the phase-encoded design, has enabled more accurate estimates of visual field maps as well as other neuronal population properties (Dumoulin & Wandell [2008]; also see Larsson & Heeger [2006] for an earlier variant of the more modern pRF modeling approach), including receptive field size and laterality. The term ‘receptive field’ was first used by C. S. Sherrington to describe the skin region from which a scratch reflex could be elicited (Sherrington, 1906; Sherrington, 1910)—i.e., the receptive field of the scratch reflex (for a more detailed account on the concepts behind pRF modeling, see Wandell & Winawer [2015]). Later, Hartline (1938) adapted the term to designate the region of the retina that must be stimulated to obtain a response in any given optic nerve fiber. Expanding on this idea and on earlier fMRI studies in humans (Smith, Singh, Williams, & Greenlee, 2001; Tootell et al., 1997), the pRF represents the collective receptive field properties of a population of neurons within a given spatial unit (typically a voxel, the smallest volumetric unit of fMRI data). In this context, pRF properties capture how the voxel's constituent neurons respond to sensory stimuli, encompassing various aspects of neuronal tuning and selectivity.

**Figure 2.**
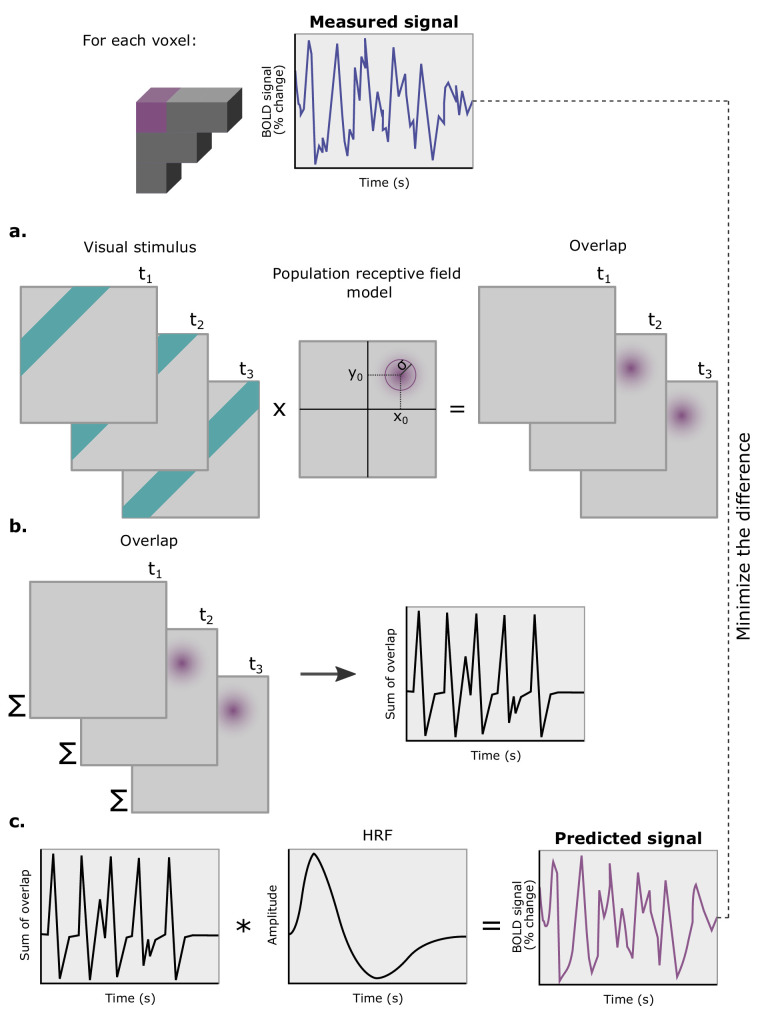
pRF modeling. **The p**RF models are estimated for each voxel independently. (**a**) The overlap between the visual stimulus and the pRF model is determined. This parameterized model of the pRF is a 2D isotropic Gaussian, with three parameters: *x*_0_ and *y*_0_, the center of the 2D Gaussian, or the pRF center location; and *σ*, the Gaussian spread, or pRF size. (**b**) Spatial summation of the overlap between the visual stimulus and the pRF model is calculated for each time point. (**c**) The resulting temporal signal is convolved with a model of the hemodynamic response function (HRF), yielding the predicted signal. Note that the pRF parameters are estimated to minimize the difference between the predicted and the measured fMRI signals.

Whereas phase-encoded methods only yield an “average” pRF center (Engel, 2012), pRF modeling characterizes pRFs as distributions, directly linking fMRI signals to neuronal population properties. By refining how visual field representations are mapped, pRF modeling has significantly enhanced the precision of retinotopic measurements. Briefly, pRF mapping models the fMRI signal as a linear function of the portion of the visual stimulus that overlaps with a parameterized model of the pRF at a given location in the visual field (Figure 2). The pRF is typically modeled as a 2D isotropic Gaussian function with three parameters: *x*_0_ and *y*_0_, which define the center of the 2D Gaussian (i.e., the pRF center location), and *σ*, which represents the Gaussian spread, or the pRF size. These parameters are typically optimized to minimize the discrepancy between the predicted and observed fMRI signals through a two-stage process. Initially, rough estimates of the pRF parameters are generated through a grid search over thousands of different modeled fMRI time series. In the second stage, the best-fit parameters are fine tuned using a nonlinear optimization algorithm, resulting in a more precise estimate of the pRF.

pRF models have numerous strengths compared with phase-encoded models. One primary strength is their ability to estimate pRF size, which increases with eccentricity in the early visual cortex and along the visual cortex hierarchy. Phase-encoding models in comparison cannot estimate pRF size, and those properties that they can estimate (i.e., spatial location) are less accurate than pRF model-based estimates (Alvarez, De Haas, Clark, Rees, & Schwarzkopf, 2015; Dumoulin & Wandell, 2008). Another strength of pRF models is their flexibility, which allows them to accommodate a wide variety of stimulus configurations, including rings, wedges, sweeping bars, and logarithmically scaled stimuli, all of which provide unique advantages for capturing receptive field properties across different eccentricities (Chang, Fine, & Boynton, 2025; Linhardt et al., 2021). For example, the use of sweeping bars as visual stimuli allows pRF models to more effectively capture the detailed spatial representations in peripheral vision, where traditional designs (e.g., rings and wedges only) might be less effective in providing sufficient resolution (Linhardt et al., 2021). Additionally, pRF modeling can be adapted with alternative parameterizations beyond the standard 2D Gaussian, such as different pRF shapes (Greene, Dumoulin, Harvey, & Ress, 2014; Merkel, Hopf, & Schoenfeld, 2018; Silson, Reynolds, Kravitz, & Baker, 2018), broadening its potential for diverse application—although some challenges and limitations exist with these adaptations (Lerma-Usabiaga, Winawer, & Wandell, 2021).

Building on this flexibility, pRF modeling has been integrated into a Bayesian framework, allowing for the incorporation of prior knowledge and uncertainty into the estimation process. This approach not only improves model accuracy and robustness, but also provides a principled way to explore the range of plausible solutions and quantify the uncertainty surrounding them (Zeidman, Silson, Schwarzkopf, Baker, & Penny, 2018). This framework provides a more flexible approach to pRF modeling, enabling the estimation of receptive fields in contexts where data might be sparse or noisy. pRF modeling has also been adapted to estimate spatiotemporal pRFs, where the temporal signal obtained from step ii in Figure 2 is convolved with a temporal response function before proceeding to step iii (Kim, Kupers, Lerma-Usabiaga, & Grill-Spector, 2024; Kupers, Kim, & Grill-Spector, 2024). This extension of pRF modeling allows for a more nuanced analysis of dynamic neural responses over time. Furthermore, new algorithms have been developed to reduce estimation time, contributing to the broader applicability of pRF mapping in both research and clinical settings (Bhat, Lührs, Goebel, & Senden, 2021; Mittal, Woletz, Linhardt, & Windischberger, 2025).

As these advancements continue to expand the capabilities of pRF modeling, its applications have broadened significantly. Since its introduction, pRF modeling has been widely used for retinotopic (Alvarez et al., 2021; Benson et al., 2018; van Es, van der Zwaag, & Knapen, 2019; Zeidman et al., 2018), somatotopic (Puckett, Bollmann, Junday, Barth, & Cunnington, 2020; Schellekens, Petridou, & Ramsey, 2018; Wang et al., 2021), and tonotopic mapping (Allen, Mesik, Kay, & Oxenham, 2022); for examining the effects of visual attention (Kay, Weiner, & Grill-Spector, 2015; Klein, Harvey, & Dumoulin, 2014; Klein et al. 2018; Puckett & DeYoe, 2015); to investigate the biological basis of clinical conditions (Brewer & Barton, 2012; Dumoulin & Knapen, 2018; Muckli, Naumer, & Singer, 2009); and more (for a reviews in this topic, please refer to Wandell & Winawer, 2011; Wandell & Winawer, 2015). Like the phase-encoded design, pRF modeling has surpassed its initial purpose in retinotopic mapping, contributing significantly to the broader neuroimaging community.

### Alternatives to pRF modeling

Beyond pRF modeling and phase-encoded designs, there are now a few alternative methods for estimating retinotopic maps. For instance, connective field modeling is an adaptation of the traditional pRF modeling, in which fMRI signals are modeled as a function of the activity in another part of the brain (neural-referred) (Haak et al., 2013), as opposed to the stimulus-referred pRF modeling approach (Dumoulin & Wandell, 2008). In other words, localized activity in one cortical region, such as V1, elicits responses in voxels in other cortical regions (Harvey & Dumoulin, 2011; Heinzle, Kahnt, & Haynes, 2011), for example, V2 and V3. A clear advantage of connective field modeling, in comparison with traditional pRF modelling, is its ability to accommodate a wider range of experimental paradigms (Knapen, 2021), such as resting-state or free-viewing movie-watching experiments. Specifically, if retinotopic maps in V1 are predetermined (which can be reliably predicted in most neurologically typical individuals, as we will discuss in the following section), it becomes possible to estimate retinotopic organization throughout the brain. This approach holds particular promise for scanning and estimating retinotopic maps in patients and individuals who may struggle to remain still in the scanner, where traditional methods face limitations (Tangtartharakul, Morgan, Rushton, & Schwarzkopf, 2023). Nonetheless, the efficacy of this approach is contingent upon the precision of the assumed V1 retinotopic layout, and its applicability may be constrained in populations exhibiting atypical cortical organization, where canonical models of V1 mapping fail to accurately capture interindividual variability.

Although not as widely adopted as pRF and connective field modeling, a few modern alternatives for estimating retinotopic maps are worth highlighting. These include microprobing (Carvalho et al., 2020), topological receptive field modeling (Tu, Ta, Lu, & Wang, 2021), and alternating block design mapping (Ellis et al., 2021). Note, however, that this is not an exhaustive list, but rather a selection of methods that have been noted in the literature. Among these alternatives, microprobing is another adaptation of the traditional pRF modeling that makes minimal *a priori* assumptions about the number of pRFs per voxel (which is typically assumed to be equal to one in traditional pRF modeling) and the pRF's shape (although, as we mentioned previously, it is possible to use different pRF shapes under the pRF modeling framework). This method is particularly advantageous for mapping retinotopic organization in clinical cases. For example, in individuals with albinism, V1 exhibits an overlapping representation of the contralateral and ipsilateral hemifields owing to excessive decussation at the optic chiasm (Hoffmann, Tolhurst, Moore, & Morland, 2003). This means that voxels within V1 can contain neuronal subpopulations with receptive fields corresponding with different regions of visual space (Duwell, Woertz, Mathis, Carroll, & DeYoe, 2021; Kaule et al., 2014), which is not captured by traditional pRF modeling (Carvalho et al., 2020). Topological receptive field modeling enforces topological constraints to ensure the continuity of visual field maps within visual areas by optimizing a topology-preserving function (Tu et al., 2021). A potential advantage of this approach is that it reconstructs smooth retinotopic maps, making it easier to identify features such as isoeccentricity lines, which are crucial for studying cortical magnification but difficult to define in noisy empirical maps. The alternating block design, as implemented by Ellis at al., (2021), offers an alternative to pRF modeling and the phase-encoded design for retinotopic mapping in human infants by presenting meridian or spatial frequency stimuli in a temporally alternating block design. The meridian stimulus is designed to reveal polar angle reversals necessary to delineate visual area boundaries, also termed as meridian mapping (Grill-Spector, Kushnir, Edelman, Itzchak, & Malach, 1998; Grill-Spector et al., 1998), whereas the spatial frequency stimulus reveals coarse-scale eccentricity maps (Arcaro & Livingstone, 2017; Henriksson, Nurminen, Hyvärinen, & Vanni, 2008). This simplified methodology may be more tolerant to eye movement because the visual stimulation is mostly uniform across the visual field, making it suitable for awake, behaving infants.

## Beyond measurement: Computational models for predicting retinotopy

Although estimating retinotopic maps has traditionally relied on empirical fMRI data (i.e., measurement), there is substantial interest in predicting these maps through computational models owing to the high scanning cost, time, and expertise required to measure them. Such models of retinotopy are fundamentally grounded in studies showing that retinotopic organization adheres to predictable anatomical patterns (Hinds et al., 2008; Horton & Hoyt, 1991; Inouye, 1909; Lister & Holmes, 1916; Rajimehr & Tootell, 2009), suggesting that functional organization can be inferred from anatomy to some extent. Moreover, advances in cortical surface reconstruction (Dale, Fischl, & Sereno, 1999; Despotović, Goossens, & Philips, 2015; Wandell, Chial, & Backus, 2000) have played a crucial role in enabling the development of these models, providing the necessary framework for visualizing and representing cortical organization, because they preserve the spatial relationships within and between visual areas. The anatomical principles revealed by previous studies have, therefore, laid the foundation for these computational models to predict retinotopic organization from anatomy, offering a powerful alternative when functional data is unavailable or of insufficient quality (e.g., in historical data from healthy participants). These models not only serve as a valuable tool when functional data are unavailable, but also provide an exciting opportunity to explore interindividual variability in retinotopy, revealing how structural differences may relate to differences in brain function and perception. Therefore, we now turn our attention to the fundamentals of these models, their potential applications, and the novel insights they can offer.

### Close-form models

**Figure 3.**
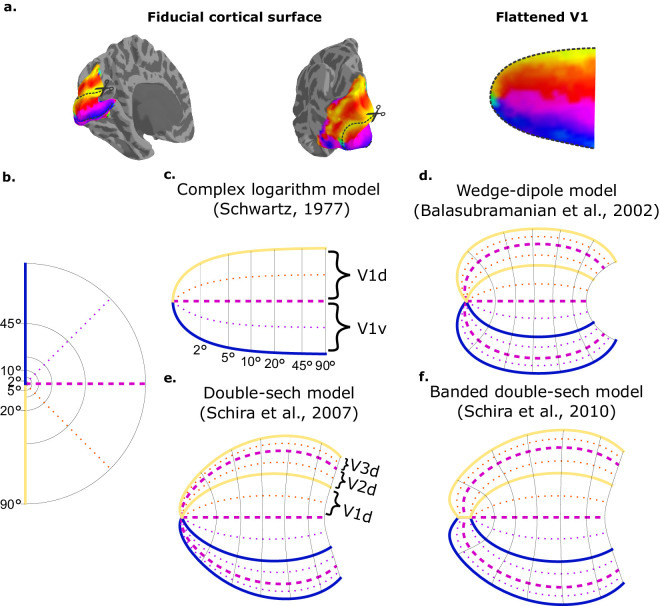
Closed-form models of retinotopic organization in human visual cortex. (**a**) Polar angle representation in a fiducial model of the cortical surface (left and middle) with V1 indicated by the dashed grey lines. The flattened (or unfolded) representation of V1 is shown on the right. (**b**) Right visual hemifield representation in polar coordinates. (**c**) Complex logarithm model proposed by Schwartz (1997). A logarithm transformation is applied to visual field coordinates, represented as complex numbers. Radial lines (indicated by different colors) and concentric circles (at 2°, 5°**,** 10°, 20°, 45°, and 90° of eccentricity) in the visual field are mapped onto horizontal and vertical lines in the unfolded primary visual cortex. (**d**) The wedge-dipole model introduces an intermediate representation of the visual field, the wedge map, to capture the retinotopic organization of V1, V2, and V3. (**e**) The double-sech model introduces two shear functions to the dipole model (as in c) to represent meridional isotropy (i.e., equal areas of the visual field are mapped to equal areas of the cortex) of areal magnification. (**f**) Schira et al. (2010) extended the previous model (as in d) by modifying the intermediate wedge representation in the center of the visual field to account for the banded foveal representation (Schira et al., 2009; Schira et al., 2010).

The link between retinotopic organization and anatomical structure was recognized in the early 20th century (Inouye, 1909; Lister & Holmes, 1916). Although there was some intuition that a mathematical description of the projection of the visual field onto the cerebral cortex based on anatomical landmarks might exist (Polyak, 1932), the first formal mathematical formulation did not emerge until the 1970s (Schwartz, 1977). Schwartz (1977) took inspiration from the mathematical definition of linear cortical magnification—the ratio of the change in cortical position to the related change in visual field position in units of mm/deg and expressed as *m(r) = k/r*, to propose an expression for the visual field projection, a function whose derivative is radially symmetric and proportional to 1/*r*, like the linear cortical magnification expression. A function that could satisfy both conditions is the complex logarithm, a generalization of the natural logarithm to complex numbers, where the complex number *r + θi* express visual field coordinates (*r*, θ). Simply put, the complex logarithm transforms radial lines and concentric circles in the visual field (Figure 3b) into “horizontal” and “vertical” lines (Figure 3c), just like the visual field projection in the unfolded (or flattened) primary visual cortex (Figure 3a).

Although the complex logarithm model—also referred to as the monopole model—captured the shape of the flattened V1 (Figures 3a and 3c) and other details of its retinotopic organization in parafoveal regions (near the center of vision), it failed to represent the shape of V1 at high eccentricities accurately (the visual periphery) based on available empirical data from non-human primates (Schwartz, 1984). To address this limitation, Schwartz (1984) proposed an improved model, the dipole model, which better represented retinotopic maps at high eccentricities. This formulation was later adapted to capture the retinotopic organization of V1, V2, and V3 (Balasubramanian, Polimeni, & Schwartz, 2002), known as the wedge-dipole model (Figure 3d), by using an alternate wedge representation of the visual field.

Although these models provided a mathematical framework to describe the general appearance of retinotopic maps, they were not rigorously compared with empirical data. For instance, neither the monopole model nor the dipole model accounted for the observation that, at a given eccentricity, equal areas of the visual field are mapped to equal areas of the cortex—a property known as meridional isotropy of cortical magnification (Schira, Wade, & Tyler, 2007). To address this, Schira et al. proposed the Double-Sech model (Figure 3e) by modifying the dipole model with the introduction of two shear functions. This adaptation allowed the model to better represent meridional isotropy of areal magnification in V1, aligning it more closely with empirical findings (Schira et al., 2007). Note, however, that recent studies examining magnification around the visual field have observed some asymmetries in cortical magnification along a given eccentricity (Benson, Kupers, Barbot, Carrasco, & Winawer, 2021; Silva et al., 2018). These recent studies have likely observed these asymmetries owing to increased sample sizes and to methods specifically developed to measure magnification in the tangential direction.

The Double-Sech model was soon followed by the banded Double-Sech model (Schira, Tyler, Spehar, & Breakspear, 2010). This revised model considered the latest finding that the foveal confluence was represented as multiple bands or strips across V1/V2/V3 in the human visual cortex (Schira, Tyler, Breakspear, & Spehar, 2009) instead of a single (converging) point shared between V1/V2/V3. For the revised model, besides introducing shear functions to the dipole model (Schira et al., 2007), they used a modified wedge representation of the visual field (Balasubramanian et al., 2002) to capture the topology of V1/V2/V3 retinotopic organization as in the wedge-dipole model (Figure 3f). Specifically, the wedge representation was slightly modified in the center of the visual field to account for the banded foveal representation in early visual areas.

**Figure 4.**
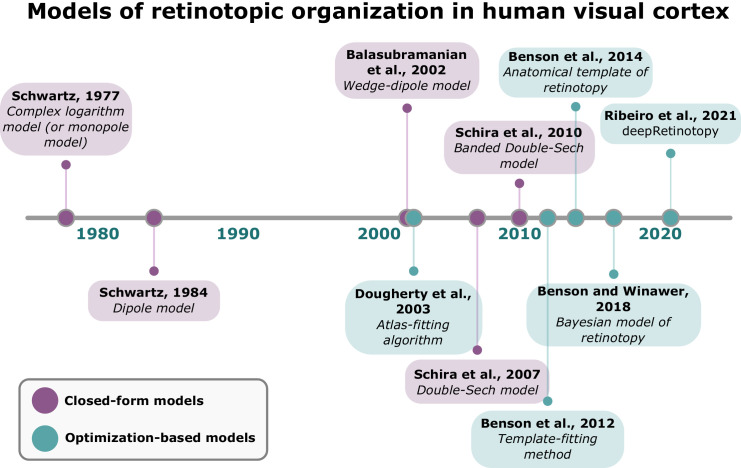
A timeline of models of retinotopy over 50 years. All models explicitly discussed herein are shown in a timeline. The closed-form models are in purple, and in green are the optimization-based models. The model names are as per the original scientific articles, but for simplicity, atlas and template are used as synonyms throughout this article.

Together, these closed-form models have been instrumental in revealing the mathematical principles underlying retinotopic organization. All the retinotopy models discussed thus far are highlighted in purple in Figure 4, whereas subsequent work—consisting of optimization-based approaches to predict individual-level retinotopic maps from cortical anatomy—is shown in green. Unlike closed-form models, optimization-based models accommodate more parameters with fewer assumptions. Accordingly, a model with fewer parameters may fail to capture the true underlying retinotopic organization and instead predict an average retinotopic map (high bias). In contrast, having too many parameters may lead to potential overfit to empirical noise (high variance). Although optimization-based models may allow for the prediction of interindividual variability in retinotopic organization, the bias–variance trade-off must be considered, especially when it is still unknown whether the observed variability in retinotopic organization is neurogenic. The following section explores these optimization-based models in greater detail.

### Optimization-based models

The first optimization-based model of retinotopy in humans was an atlas-fitting algorithm for 2D flat maps (Dougherty et al., 2003) that applies a non-linear co-registration technique to minimize the difference between the empirical retinotopic maps and a template of the expected retinotopic organization of V1/V2/V3. The optimal deformation was determined through an iterative process to minimize the error (difference between empirical and template map) and amount of deformation applied. A crucial assumption from this method is that true retinotopic organization is smooth and continuous, and discontinuities seen in empirical maps would likely reflect noise in the data (Dougherty et al., 2003). Therefore, as the authors suggest, fitting a smooth and continuous template would likely be a better indicator of the retinotopic organization in an individual's visual cortex than their empirical ‘noisy map’. A key advantage of this procedure is allowing the researcher to define specific points or strips (e.g., isoeccentricity lines) within the retinotopic maps, which are difficult to define on empirical noisy maps, but are crucial for the study of properties like cortical magnification. Despite that, and similarly to the closed-form models, the method was constrained to 2D (or flattened) representations of the early visual areas, with inherent distortions to the spatial relationship between adjacent areas.

**Figure 5.**
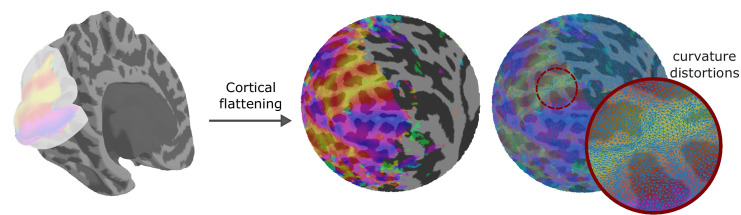
Cortical surface flattening introduces curvature distortions. The cortical surface representation (left) facilitates the visualization of brain activity while preserving the natural spatial relationships between adjacent neuronal populations. However, it conceals areas buried within sulci. Flattening the cortex reveals these hidden regions (middle) but introduces distortions in spatial relationships (right). The inset image shows a magnified portion of the polar angle map where the surface mesh has been overlaid upon (in blue). The distortions introduced by cortical flattening can be seen as triangles being more compressed along the gyrus compared with triangles along the sulci.

Accordingly, although cortical flattening facilitates visual inspection of brain activity distributed along the cortical surface as it reveals relationships between areas buried within sulci, it also introduces distortions. Typically, surface flattening involves unfolding the ‘crumpled’ cortical surface by introducing cuts, while preserving the distance between points from the initial (folded) representation as best as possible (Figure 5). However, unfolding convoluted surfaces, with many bumps like gyri, introduce distortions to the spatial relationship between adjacent areas. Notably, protuberant regions are compressed on the unfolded representation, posing challenges in accurately preserving the cortical landscape's details [for an in-depth discussion about distortions introduced by cortical flattening, see Wandell et al. (2000)].

Iterating on the atlas-fitting algorithm (Dougherty et al., 2003), Benson et al. (2012) proposed a template-fitting method in which individual-level predictions are determined by registering the individual's cortical anatomy to an anatomical atlas (Fischl, Sereno, Tootell, & Dale, 1999) on which a model of V1 retinotopic organization was defined. This work built on previous studies that demonstrated the close relationship between the functional organization of V1 and the underlying structure (Hinds et al., 2008; Horton & Hoyt, 1991; Inouye, 1909; Lister & Holmes, 1916; Rajimehr & Tootell, 2009) by defining the functional model directly in terms of the anatomy. Because the template-fitting method relies only on the registration of individual subject structural data to a template, it can generate retinotopic predictions at the individual-level in the absence of functional measurements (Benson et al., 2012).

This template-fitting method was later extended to V2 and V3 (Benson, Butt, Brainard, & Aguirre, 2014; referred to as the anatomical template of retinotopy) by adapting the atlas-fitting method (Dougherty et al., 2003) and incorporating the closed-form model of Schira et al. (2010). To accomplish this, individual subject data were first registered to a common anatomical template as in previous work, and pRF parameters of the retinotopic maps were averaged across subjects in this space. The averaged retinotopic maps were then registered to a closed-form model of V1, V2, and V3 retinotopic organization (Schira et al., 2010) using an error minimization algorithm similar to the registration technique of Dougherty et al. (2003). The registration of the average retinotopic map to model can then be inverted to yield the closed-form model of retinotopy in the space of the anatomical template. In other words, this method (Benson et al., 2014) essentially combines the atlas-fitting algorithm (Dougherty et al., 2003) with the anatomical alignment methods of FreeSurfer (Fischl et al., 1999) to apply closed-form models to novel subjects using their cortical anatomy only.

**Figure 6.**
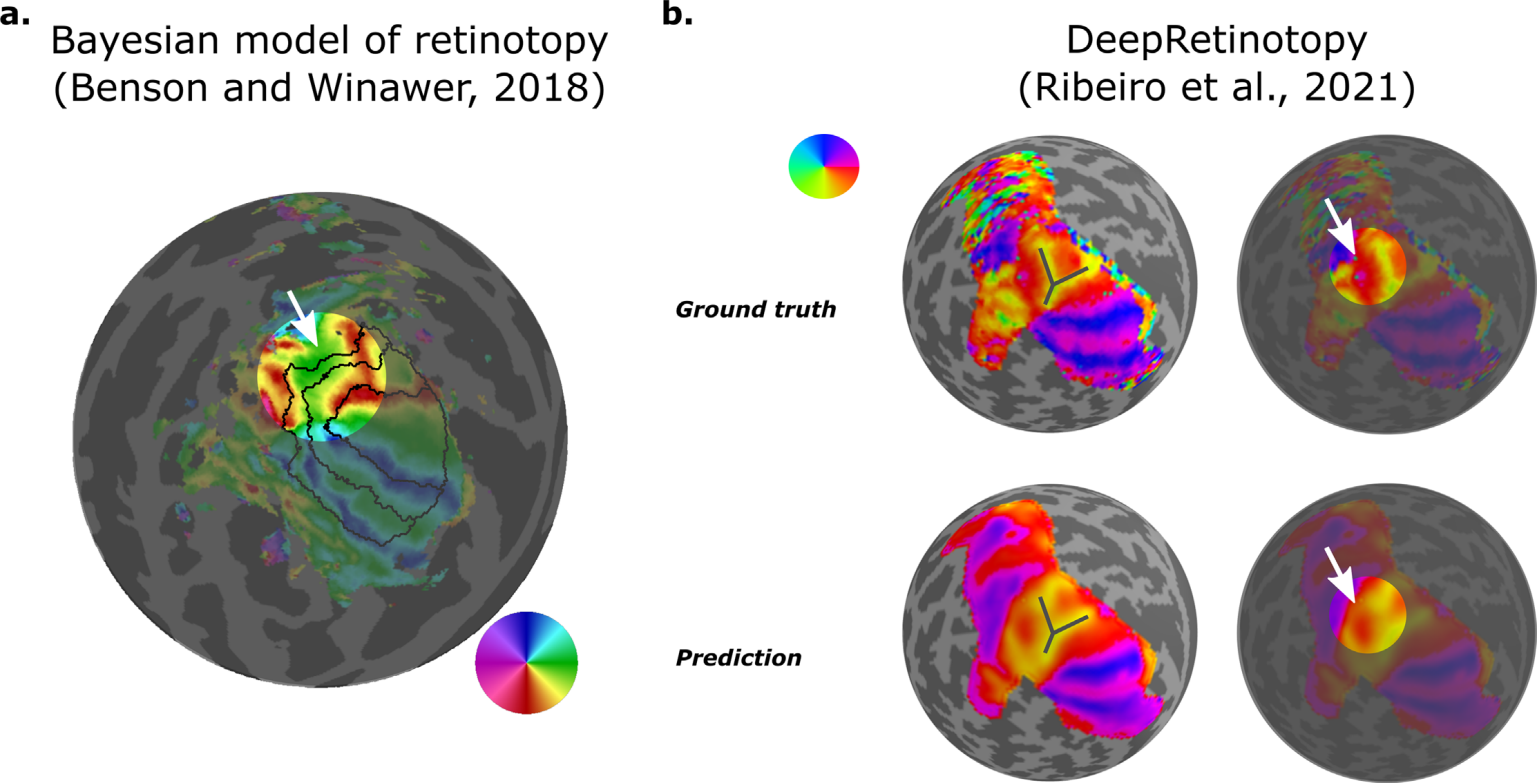
Recent optimization-based models of retinotopic organization in human visual cortex. (**a**) The panel illustrates the empirical retinotopic map and predicted boundaries (black lines) from the Bayesian model of retinotopy for one individual in the Human Connectome Project (HCP) dataset (HCP #198653) (Benson et al., 2018). In the Bayesian model of retinotopy, a prior (a template) and an observation (some minutes of functional data) are combined to generate individual-level retinotopic maps (Benson & Winawer, 2018). However, this model cannot predict discontinuities in the lower vertical meridian representation seen in the dorsal portion of early visual cortex of some individuals**—**for example, see the white arrow: note that a continuous lower vertical meridian would appear as an unbroken red color following the dorsal border of V3, rather than being interrupted by yellow/green as seen here. (**b**) DeepRetinotopy, a geometric deep learning model, was trained to predict retinotopic maps from anatomical information (curvature and myelin maps) on a cortical surface model of the human brain. Similarly, the panel illustrates the ground truth (empirical) and predicted maps for the same participant (HCP #198653) with and without grey lines and white arrows indicating the unique polar angle representation. Note that deepRetinotopy was able to predict the aforementioned discontinuity.

More recently, Benson and Winawer (2018) proposed a Bayesian model of retinotopy to better capture interindividual variability in retinotopic organization by using a small amount of empirical mapping data. Within this Bayesian framework, a prior (a retinotopic map model defined on an anatomical template) and an observation (some minutes of functional data) are combined to generate individual-level retinotopic maps. By incorporating functional data, this approach was able to improve predictions of left out retinotopy data compared with previous anatomy-only methods (Benson et al., 2012, Benson et al., 2014). Additionally, when anatomy is controlled, it produces quantitative estimates of the similarity between retinotopic maps (across individuals and between empirical and predicted maps). However, although this approach can predict some level of interindividual variability, it cannot do so from anatomical information alone. Crucially, because this model assumes a probability of zero to any map that differs topologically from the retinotopic map template (prior), this model is unable to predict certain idiosyncrasies, e.g., discontinuities in the lower vertical meridian representation seen in the dorsal portion of early visual cortex in some individuals (Figure 6a).

Although these optimization-based models have seen wide adoption over empirical models owing to their clear usefulness (Albers, Meindertsma, Toni, & de Lange, 2018; Alvarez, Parker, & Bridge, 2019; Cichy, Khosla, Pantazis, Torralba, & Oliva, 2016; Colizoli, de Gee, van der Zwaag, & Donner, 2022; Isherwood, Schira, & Spehar, 2017; Müller-Axt, Anwander, & von Kriegstein, 2017; Rosenke et al., 2018; Storrs, Kietzmann, Walther, Mehrer, & Kriegeskorte, 2021), such models are unable to account for any idiosyncratic differences in the structure–function relationship from anatomical information alone due to their initial assumption of a template—that is, a rigid topology. Importantly, once anatomical differences have been normalized, individual-level predictions are topologically identical since the template is projected to a common cortical surface space, to which individuals’ cortical anatomies are registered (Benson et al., 2014). Even the Bayesian model of retinotopy, which predicts more interindividual variability by leveraging a few minutes of functional data, is unable to predict idiosyncrasies in the topological arrangement of retinotopic maps owing to its reliance on a prior. Evidence suggests, however, that there may be significant interindividual variability in non-human primates (Angelucci & Rosa, 2015; Gattass, Sousa, & Gross, 1988; Zhu & Vanduffel, 2019) as well as in humans (Allen et al., 2021; Arcaro & Kastner, 2015; Benson et al., 2014; Benson & Winawer, 2018; Ribeiro et al., 2023; Van Essen & Glasser, 2018).

Given these limitations, an alternative approach involves a learning mechanism that determines a mapping function for predicting retinotopy from anatomy without relying on a rigid topological template. Through supervised learning, a deep learning model could infer this structure–function relationship directly from data. Ribeiro, Bollmann, and Puckett (2021) implemented this concept with deepRetinotopy, a model that predicts individual-level retinotopic organization using anatomical data represented on the curved cortical surface, thereby avoiding distortions introduced by cortical flattening.

Leveraging the size of the Human Connectome Project 7T Retinotopy dataset (*n* = 181) (Benson et al., 2018), Ribeiro et al. (2021) developed a geometric deep learning model—a model specifically designed to handle non-Euclidean data structures (Bronstein, Bruna, Lecun, Szlam, & Vandergheynst, 2017)—to learn the structure–function relationship in the human visual cortex. Models were trained to predict retinotopic maps from anatomical information (curvature and myelin maps) represented on a cortical surface model of the human brain. These models were able to accurately predict retinotopic organization not only in early visual areas (V1–V3), where prediction confidence is particularly high, but also across a broad array of 18 additional higher-order visual areas (Wang et al., 2015), showcasing substantial spatial coverage (see Future Directions for a caveat). In addition to this breadth, the deepRetinotopy models captured fine-grained individual differences in polar angle representations (Figure 6b), including the previously unmodeled discontinuities in the lower vertical meridian representation within dorsal early visual cortex, an advance over earlier models of retinotopy.

Although models of retinotopy continue to improve in accuracy, existing approaches have enabled the prediction of individual retinotopic organization from anatomical data, reducing reliance on fMRI acquisition and processing while facilitating large-scale analyses of interindividual variability. Although direct retinotopic mapping remains the most precise method for individual-level analysis, variations in data processing, pRF implementations, and visual stimuli can introduce discrepancies in estimates (Himmelberg et al., 2021; Linhardt, Woletz, Paz-Alonso, Windischberger, & Lerma-Usabiaga, 2025). In contrast, computational models provide a standardized framework for cross-study and cross-individual comparisons. In the following section, we explore the potential of these models to deepen our understanding of interindividual variability, enhance predictive accuracy, and uncover links between retinotopic organization and human perception.

## Future directions

### Interindividual variability

Interindividual variability in the functional organization of the visual cortex is evident not only in differences in the size and spatial location of visual areas (Amunts et al., 2000; Benson et al., 2022; Dougherty et al., 2003; Hasnain et al., 1998; Rademacher et al., 1993; Stensaas et al., 1974), but also in variations in the topology of retinotopic maps. For example, studies have suggested that the dorsal third-tier visual cortex may exhibit a more complex organization, with topological differences observed in both non-human primates (Angelucci & Rosa, 2015; Gattass et al., 1988; Sereno, McDonald, & Allman, 2015; Zhu & Vanduffel, 2019) and humans (Arcaro & Kastner, 2015; Benson & Winawer, 2018; Van Essen & Glasser, 2018). However, these findings are often dismissed as artifacts, particularly in human studies, in part owing to limited sample sizes.

Building on these earlier suggestions, Ribeiro et al. (2023) used the large-scale HCP 7T Retinotopy dataset (Benson et al., 2018) to systematically investigate interindividual variability in retinotopic maps. This study provided more robust evidence that the retinotopic organization is more variable than previously assumed. Notably, greater variability was found in the dorsal visual areas compared with the ventral areas, with left-hemisphere maps exhibiting more variability than those in the right hemisphere. Detailed visual field sign analyses (Sereno, McDonald, & Allman, 1994; Sereno et al., 1995) also revealed a shared discontinuity in the region traditionally identified as dorsal V3, reinforcing the notion of a more diverse retinotopic organization in this area (Arcaro & Kastner, 2015; Benson & Winawer, 2018; Van Essen & Glasser, 2018).

Although these studies offer valuable insights into the interindividual variability of retinotopic maps, further research is necessary to determine whether these differences reflect genuine biological variability or result from measurement errors (e.g., related to anatomical folding patterns). Computational models of retinotopy present a promising solution to this challenge, offering a standardized framework for identifying sources of variability. For instance, explainability techniques can pinpoint which input features contribute to differences in predicted retinotopic maps (Ribeiro, Bollmann, Cunnington, & Puckett, 2022). These insights can then guide targeted analyses to detect potential artifacts, such as cortical surface tessellation errors or the influence of blood vessels on retinotopic map reconstruction in localized areas. Thus, computational models of retinotopy can help detect individuals with atypical maps, enabling follow-up investigations to distinguish between artifacts and genuine biological differences.

Understanding these individual differences in retinotopic organization is critical to advancing our understanding of visual processing and neurobiology. Variability in how the brain organizes visual information may influence perceptual abilities, cognitive functions, and responses to sensory stimuli, making it essential to account for such variability in both basic and applied neurobiological research.

### Structure–function relationship

Beyond offering a valuable framework for studying retinotopic organization at the individual level, computational models also underscore the limitations of assuming a single, rigid template for predicting retinotopic maps, highlighting the need for a deeper understanding of how structure and function interact across individuals. Retinotopic maps are generally consistent across individuals, and while the mapping function between the visual field and the cortex (at least in V1) is often captured by relatively simple closed-form models, this approach does not apply to higher-order visual areas. For example, discontinuities in the functional organization of the dorsal portion of early visual areas (Figure 6) suggest that interindividual variability in the underlying structure–function relationship cannot be captured by a rigid topological representation. By learning this relationship from the data, Ribeiro et al. (2021) demonstrated that deep learning models can flexibly adapt to an individual's anatomy, generating idiosyncratic functional maps. These findings further highlight that a one-size-fits-all approach—that is, using a single fixed topological template of retinotopic organization—overlooks meaningful interindividual differences. Future work may seek to characterize variability in retinotopic maps’ size, location, and topology and establish biological correlates of such variability. Such research could ultimately identify common organizational motifs in retinotopy, paving the way for the development of more comprehensive templates that better capture individual differences.

Beyond capturing interindividual variability in early visual areas, computational models also provide a tool for evaluating the strength of structure–function coupling across the visual cortex. While most previous models focused on early visual areas, deepRetinotopy (Ribeiro et al., 2021) and the Bayesian model of retinotopy (Benson & Winawer, 2018) have extended predictions to higher-order visual areas (note that Benson & Winawer used areas beyond V1–V3 to stabilize the Bayesian inference method, without claiming accurate predictions). However, both studies found higher prediction errors in these regions compared with early visual cortex. Owing to the lower reliability of empirical retinotopic mapping data in higher-order areas, it remains unclear whether these errors reflect poor data quality (e.g., from stimuli that are poorly designed for stimulating later visual areas) or a genuinely weaker structure–function coupling. Future studies could leverage improved empirical data, or additional evidence for the location of higher order areas derived from connective field modeling (Haak et al., 2013), to systematically assess how data quality affects model predictions. For instance, if models trained on higher-quality data fail to achieve better predictions, this would suggest that functional organization in higher-order visual areas is more decoupled from cortical anatomy.

Refining these models further will not only help to resolve such uncertainties, but also provide deeper insights into the structural constraints shaping functional organization across individuals. By integrating multimodal imaging data, such as diffusion MRI to map white matter connectivity or myelin-sensitive imaging to assess cortical microstructure, future models could better capture the biological underpinnings of retinotopic organization. Additionally, explainability techniques could be leveraged to reveal which anatomical features most strongly influence predicted retinotopic maps, shedding light on whether variability in structure–function coupling is driven by individual differences in cortical architecture or by higher-order functional demands. Ultimately, these approaches will be crucial in advancing our understanding of how the brain's structural and functional organization interact to support visual processing, with potential applications in both basic neuroscience and clinical interventions for visual disorders.

### Brain function and behavior

Although understanding structure–function relationships is crucial for linking brain organization to function, it is equally important to explore how variability in both brain structure and functional organization translates into individual differences in human behavior (Genon, Eickhoff, & Kharabian, 2022; Himmelberg, Winawer, & Carrasco, 2023; Kanai & Rees, 2011; Seghier & Price, 2018; Vogel & Awh, 2008). For instance, the surface area of V1 varies by at least a factor of three across individuals—substantially more than the approximately 1.5-fold variation observed in total neocortical surface area (Benson et al., 2022; Dougherty et al., 2003). This pronounced variability makes V1 an important target for studies examining structure–function relationships. Previous studies have shown that the size or, similarly, the cortical magnification, of V1 is associated with the subjective experience of object size (Schwarzkopf & Rees, 2013; Schwarzkopf, Song, & Rees, 2011), Vernier acuity (Duncan & Boynton, 2003), perceptual and orientation discrimination (Song, Schwarzkopf, Kanai, & Rees, 2015; Song, Schwarzkopf, & Rees, 2013), contrast sensitivity (Benson et al., 2021; Himmelberg, Winawer, & Carrasco, 2022), visual working memory (Bergmann, Genç, Kohler, Singer, & Pearson, 2016a), and mental imagery (Bergmann, Genç, Kohler, Singer, & Pearson, 2016b). However, despite this significant variability in V1 size, the behavioral effects associated with it tend to be relatively small, raising the question of why a threefold difference in cortical surface area does not lead to correspondingly large differences in visual function. More broadly, beyond V1, clear structure–function associations remain less well established (He, Wang, & Fang, 2019; Kurzawski, Pelli, & Winawer, 2021; Kurzawski et al., 2023).

A potential reason for this gap is the difficulty of obtaining detailed retinotopic maps in large samples. Computational models of retinotopy provide a promising approach to address this challenge, offering a principled way to examine how individual differences in cortical organization relate to behavior. If these models can reliably predict pRF parameters and delineate visual areas, they could be applied to large, diverse neuroimaging datasets that lack direct retinotopic mapping data, enabling broader investigations into the relationships between brain structure and function, and their ultimate connection to behavior. Furthermore, the application of computational models goes beyond just mapping the structure–function relationship. These models can be refined to predict how individual differences in cortical organization influence behavior across different contexts or in response to environmental changes. Specifically, these models may be leveraged in a normative modeling framework for understanding differences at the level of a single individual (Segal et al., 2025), where deviations from the expected retinotopic organization (that is, predicted from models of retinotopy) can be used to predict behavioral changes over time, such as during learning or in response to neuroplastic changes (Wandell & Smirnakis, 2009).

### Improving prediction accuracy

Having established the importance of improving computational models to better understand interindividual variability and structure–function relationships, it is essential to consider how these models can be refined further to enhance prediction accuracy. Although advances in computational modeling hold great promise for improving our understanding of individual differences in brain function and behavior, the accuracy of these models is ultimately constrained by the quality of empirical data (see Liu [2016] for a review) and the encoding models used to generate training data. This brings us to the crucial challenge of improving prediction accuracy.

Because of this fundamental challenge, it remains unclear to what extent interindividual differences in retinotopic organization arise from methodological factors versus genuine biological variation. Regardless of the source, a deeper understanding of measurement noise in retinotopic mapping data could enhance both the accuracy of encoding models in estimating pRF parameters and the predictive power of computational models of retinotopy significantly.

Some sources of noise—such as thermal noise, subject motion, or eye movements—are uncorrelated across scans and can be mitigated by averaging over repeated measurements. However, other noise sources persist across scans and are more difficult to address. These include the partial volume effect, where a single voxel integrates signals from distant cortical locations (Ballester, Zisserman, & Brady, 2002), and vascular drainage effects, which introduce spatial biases in the hemodynamic response (Boyd Taylor, Puckett, Isherwood, & Schira, 2019; Dagli, Ingeholm, & Haxby, 1999; Lee, Glover, & Meyer, 1995; Menon, 2002; Olman, Inati, & Heeger, 2007; Winawer, Horiguchi, Sayres, Amano, & Wandell, 2010). Although more sophisticated models of neurovascular dynamics may eventually help to mitigate these artifacts, current encoding models—such as pRF mapping—largely treat voxels in isolation, neglecting broader anatomical and physiological context. Future research could explore the relationship between vascular architecture and retinotopic map variations, clarifying the origins of interindividual variability in retinotopic organization while also improving the accuracy of both encoding models and computational models of retinotopy.

Ultimately, improving the accuracy of computational models of retinotopy will not only deepen our understanding of visual cortical organization, but also have broad implications for the study of brain function and behavior. Emerging evidence suggests that visuospatial coding (such as retinotopic maps) may serve as a structural scaffold for higher-order cognitive processes (Groen, Dekker, Knapen, & Silson, 2022), raising the prospect that such models could be extended beyond the visual cortex to inform systems-level theories of brain organization. Enhancing these models to better account for noise, methodological variability, and interindividual neurobiological differences will enable more precise and personalized predictions. In turn, this refinement could advance both clinical applications and our broader understanding of cortical architecture.

## Concluding remarks

Determining how the brain maps our senses is a major goal of neuroscience. As such, we reviewed and summarized the cumulative effort of neuroscientists to empirically measure and model the retinotopic organization of human visual cortex. Several advancements in neuroimaging have enabled the mapping of the visual field in the human brain, from data acquisition with fMRI to better experimental designs to more sophisticated neuroimaging software for data analysis and visualization. Concurrently, many models of retinotopy have been proposed over the past 50 years. These models progressed from the simple closed-form complex logarithm model to a template-fitting algorithm, and most recently, deep learning models, all aiming to describe the projection of the visual field onto the human cerebral cortex mathematically or computationally. Looking ahead, we believe computational models of retinotopy offer a generalizable framework for studying interindividual variability in retinotopic maps, structure–function coupling across the hierarchy of visual areas, and the relationships between brain function and behavior.
